# Every Four-Legged Animal is Not a Dog: Investigating Concussive Symptoms in a Non-Concussed Psychiatric Sample

**DOI:** 10.1093/arclin/acaf108

**Published:** 2025-12-08

**Authors:** Kaynaat Abrar, Brianna M Goldstein, Jeremy B Frank, Konstantine K Zakzanis

**Affiliations:** Department of Psychology, University of Toronto Scarborough, Toronto, ON M1C 1A4, Canada; Department of Psychology, McGill University, 845 Sherbrooke St W, Montreal, QC H3A 0G4, Canada; InnerQore Health, 19 Centre Street, Thornhill, ON L4J 1G1, Canada; Department of Psychology, University of Toronto Scarborough, Toronto, ON M1C 1A4, Canada

**Keywords:** Post-concussive symptoms, Head injury, Traumatic brain injury, Psychiatric disorders

## Abstract

Persistent somatic, cognitive, and psychological symptoms following an uncomplicated mild traumatic brain injury are often attributed to post-concussion syndrome. However, existing research demonstrates that non-concussed populations also report high base rates of such symptoms. In this study, archival data from 131 individuals with psychiatric diagnoses were analyzed using the Rivermead Post-Concussion Symptoms Questionnaire. Participants were classified into trauma- and anxiety-related disorders, somatic symptom and related disorders, or complex psychiatric disorders. Nonparametric tests were conducted to examine group differences in RPQ total and symptom cluster scores. Significant differences were observed across diagnostic groups, with the complex psychiatric disorders group endorsing the highest total and cluster scores. Across all psychiatric groups, mean RPQ total scores exceeded those reported in multicultural healthy controls and a mTBI sample. The present study demonstrates that PCS-like symptoms are not unique to neurological trauma but are strongly endorsed across psychiatric populations, particularly among individuals with multiple psychiatric comorbidities.

## INTRODUCTION

In the post-acute stages following an uncomplicated mild-traumatic brain injury (mTBI), many individuals report the persistence of various somatic, cognitive, and psychological symptoms that can in turn, impede on one’s everyday functioning. Such symptomatology can include dizziness, irritability, fatigue, poor memory, and headache (Boake et al., 2005). The co-occurrence of these symptoms forms the basis of post-concussion syndrome (PCS), a term officially used when such symptoms persist for greater than 3 months following a mTBI (Bigler, 2008). Given the broad and nonspecific symptomatology of PCS, research has investigated the prevalence of such symptoms among diverse populations, including healthy university students, multicultural university students, non-concussed collegiate athletes, non-mTBI patients, individuals with chronic pain, and individuals seeking psychotherapy (Asken et al., 2017; Fox et al., 1995b; Iverson & McCracken, 1997; Malik et al., 2023; Wang et al., 2006; Zakzanis & Yeung, 2011). Across these studies, results demonstrate high endorsement rates of PCS symptoms among a variety of populations, notably in healthy populations, which ultimately raises concerns around the validity of PCS diagnostic criteria. This has serious implications in both clinical and research settings, increasing the possibility of incorrect diagnoses and undermining the reliability and validity of research studies on the topic.

Prominent clinical risks of a false positive PCS diagnosis include iatrogenesis, challenges with treatment effectiveness due to expectation as etiology, and the prevalence of nocebo effects. Research suggests that PCS is an example of iatrogenesis, which is defined as symptoms, illness or injury caused by medical care or attention (Bender & Matusewicz, 2013). Iatrogenic complications have been found to be related to both medication and therapeutic or diagnostic interventions (Gellman, 2020). Furthermore, iatrogenesis is categorized as a complicating psycho-legal effect in the biopsychosocial conceptualization of poor outcomes following a mild traumatic brain injury (Young, 2020). Iatrogenic effects are further exacerbated when extreme treatment methods are used, such as holding athletes from all physical activity for lengthy periods of time rather than encouraging gradual return to activity (Broshek et al., 2014). By discouraging athletes from physical activity for an extended period, PCS symptoms such as feeling tired or sluggish may be intensified due to the lengthy inactivity (Broshek et al., 2014).

Secondly, expectation as etiology within the context of PCS occurs when common symptoms are misattributed to a prior head injury (McCrea, 2007b). In one study, participants who imagined having a mild head injury expected a specific cluster of symptoms nearly identical to those seen in PCS, demonstrating that expectations can influence experiences of symptomatology as much as an actual injury can (Mittenberg et al., 1992). Indeed, research shows that expectations of negative outcomes is associated with poor concussion-related outcomes, even in otherwise healthy individuals (Hou et al., 2011; Panayiotou et al., 2011; Snell et al., 2013; Whittaker et al., 2007). The association of common complaints with an injury raises individual expectations of long-term consequences, thus making treatment a more difficult and lengthy process (McCrea, 2007b).

Finally, the nocebo effect has been a proposed explanation for why some individuals do not follow the typical course of concussion recovery (McCrea, 2007b). The nocebo effect can be defined as a negative change in health as the result of learning negative health-related information, beliefs, or experiences (Polich et al., 2019). Simply put, the nocebo effect occurs when the expectation of sickness in turn causes the sickness (Hahn, 1997). Due to the negative effects related to the use of nocebo’s, such as worsening symptoms, research suggests that nocebo effects should be minimized in concussion-related care (Polich et al., 2019). The above outlined clinical risks of a false positive PCS diagnosis fall under social and psychological factors that partially explain the persistence of PCS symptoms (Dwyer & Katz, 2018; Silverberg & Iverson, 2011). In keeping with this understanding, more recent research suggests that adopting a network perspective provides a stronger framework for understanding persistent symptoms following an uncomplicated mTBI and for developing more effective treatment methods (Iverson, 2019).

In the context of research, participants deemed to falsely represent those who have sustained a mTBI can lead researchers, and in turn, consumers of evidence-based research (may it be a clinician or various stakeholders), astray. Here, research studies that might include some, or even the entirety of a research sample of individuals with a false positive diagnosis of mTBI, might ultimately contribute to inconsistent nonreplicable findings. Indeed, evidence-based research on the neuropsychological outcome following mTBI has been and remains a decidedly heated topic of debate. To this end, there are meta-analyses, mathematical amalgamations of reported effect sizes across studies, that have reported no significant cognitive effects beyond three months or termed post-acute period of recovery (Belanger et al., 2005; Binder et al., 1997; Frencham et al., 2005;Rohling et al., 2011). Indeed, Binder et al. (1997) reported small effect sizes on neuropsychological test measures, which suggested that the prevalence of persistent neuropsychological deficits after three months post-injury is likely to be little to none. Yet one need be mindful of ecological fallacies when drawing conclusions about individuals from group-based comparison studies in keeping with the literature cited here. There is support from other studies that report persistent symptoms and cognitive deficits on neuropsychological test measures (specifically in the domains of attention and processing speed) in the post-acute period and beyond (e.g., Bedard et al., 2020; Bernstein, 2002; Chan, 2002; Hiploylee et al., 2017; Johansson et al., 2009; Mayer et al., 2017; McInnes et al., 2017; Pertab et al., 2009; Potter et al., 2002; Solbakk et al., 1999; Vanderploeg et al., 2005). Persistent neuropsychological impairments after one-year post-injury have been reported with prevalence rates of 7% (Binder et al., 1997) to more than 40% (Alves et al., 1993). Generally, estimates have ranged from 5%–20% of patients who continue to experience persistent problems in the longer term (Cancelliere et al., 2014; Cassidy et al., 2004; Iverson, 2005). Collectively, and stated another way, the research literature on outcomes following an uncomplicated mild traumatic brain injury illustrates small effect sizes on cognitive test measures (i.e., statistical evidence of test impairment), which suggests that the maximum prevalence of persistent cognitive deficit after three months post-injury is likely to be diminutive. In keeping with the most contemporary peer-reviewed critical review of recently published studies claiming long-term neurocognitive abnormalities following an uncomplicated mTBI, Boone et al. (2024) notes:

“Given that none of the 21 studies published between 2011 and 2023 and reviewed here are methodologically adequate to document long-term cognitive abnormalities from mTBI, it is concluded that the results of the six meta-analyses from 1997 to 2011, involving dozens of studies and thousands of patients in the aggregate and demonstrating no long-term cognitive abnormalities from concussion (Belanger et al., 2005; Belanger & Vanderploeg, 2005; Binder et al., 1997; Frencham et al., 2005; Rohling et al., 2011; Schretlen & Shapiro, 2003), can continue to be relied upon regarding outcome from concussion.”

Circling back, it is possible that this heterogeneity in persistence of neuropsychological impairment reflects the inclusion of false positive mTBI participants in research studies.

### Concussion Symptoms are not Specific to mTBI

The presence of PCS-like symptoms among healthy populations has been well established over the years (Chan, 2001; Gouvier et al., 1988; Gouvier et al., 1992; Iverson & Lange, 2003; Malik et al., 2023; Wang et al., 2006; Wong et al., 1994), demonstrating the nonspecificity of PCS symptomatology. Specifically, Wang et al. (2006) found relatively high base rates of postconcussion-like symptoms among healthy university students, which is consistent with previous studies investigating healthy populations (Chan, 2001; Gouvier et al., 1988; Iverson & Lange, 2003). Zakzanis and Yeung (2011) similarly found a high base rate of PCS symptoms among a healthy multicultural sample, highlighting that both cultural and linguistic backgrounds may act as a moderator in the endorsement of PCS symptomatology. Covassin et al. (2006) conducted a study investigating sex differences in neuropsychological function and concussion symptoms, finding that both male and female collegiate athletes endorsed several mild symptoms at baseline. Subsequently, studies found that even in the absence of a diagnosed brain injury, non-concussed adolescent and collegiate athletes report frequent concussion-like symptoms, with collegiate athletes reporting clinically significant rates of such symptoms (Asken et al., 2016; Asken et al., 2017).

Furthermore, a retrospective chart review found no symptom-specific differences between mTBI and non-mTBI patients, with both groups exhibiting similar chronic post-concussion symptoms (Malik et al., 2023). Another study found similar levels of reported PCS complaints between mild traumatic brain injury claimants and other injury claimants, emphasizing that PCS complaints have little diagnostic specificity (Lees-Haley et al., 2001). Reporting of such PCS symptoms are also common among individuals experiencing various types of chronic pain (Iverson & McCracken, 1997; Smith-Seemiller et al., 2003; Snell et al., 2018). To go on, increased reporting of PCS symptoms is common among psychiatric populations as well (Fox et al., 1995b; Garden & Sullivan, 2010; Meares et al., 2008). Meares et al. (2008) identified several significant predictors of acute PCS, including previous affective or anxiety disorders. Research has also found that individuals with depression exhibit elevated rates of PCS symptomatology, highlighting the complex relationship between depression and neurological conditions (Bohnen et al., 1992; Fox et al., 1995a; Gunstad, 2004; Trahan et al., 2001). Given the extensive research on the coexistence of depressive symptoms and PCS symptoms, it is not surprising that nine out of 10 patients with depression meet the liberal self-report criteria for PCS (Iverson, 2006). More recent research has further expanded on this topic, investigating depression, anxiety, and stress as predictors of postconcussion-like symptoms, discovering that stress alone was the strongest predictor of postconcussion-like symptoms amongst a nonclinical sample (Edmed & Sullivan, 2012).

Additional research has shown that PCS-like symptoms are generally not best predicted by brain injury itself, but rather are better predicted by preinjury psychiatric problems such as anxiety (Ponsford et al., 2012). Interestingly, Donnell et al. (2012) found that among their veteran sample, only 32% of those with a history of mTBI met DSM-IV criteria for PCS, whereas 91% of those diagnosed with somatization disorder met such criteria. It is important to note that their homogenous sample consisted solely of male veterans, raising concerns about generalizability to the general population. Donnell et al. (2012) also identified the role of psychiatric comorbidities, stating that the frequency of PCS symptoms increased with the number of psychiatric conditions. They found that a history of concussion in individuals with one psychiatric comorbidity increased the frequency of PCS symptoms, however in those with two or more psychiatric comorbidities, a history of concussion only added minimally to PCS symptom frequency (Donnell et al., 2012). These findings point to the possibility that the presence of PCS symptoms may not be entirely driven by initial neurotrauma itself, but rather psychiatric conditions may play an important role in the expression of PCS symptoms. Given the nonspecificity of PCS symptomatology, it is not surprising that a brain injury is not a necessary requirement to produce PCS etiology (Iverson & Lange, 2010; McCrea, 2007a), and that while mTBI contributes to acute PCS, psychological factors likely account for, or exacerbate, any ongoing symptoms experienced (Vanderploeg et al., 2014).

### The Present Examination

To date, a more specific delineation of base rates of concussion symptoms across specific psychiatric conditions has yet to be undertaken. It is important to understand the occurrence of PCS symptomatology amongst individuals with a variety of psychiatric disorders to negate false positive diagnoses of concussion in psychiatrically impaired individuals. To this end further, understanding differences in the base rate of concussion symptom endorsement between psychiatric disorders might clarify whether there are psychiatric constructs or groups who are more prone to reporting concussive symptoms. This is important to ensure accurate diagnosis of mTBI and prevent misdiagnosis in those with psychiatric conditions. Furthermore, understanding the prevalence of PCS symptoms across psychiatric disorders might clarify how these conditions contribute to or influence the reporting of such symptoms.

A factor analysis on the Rivermead Post-Concussion Symptoms Questionnaire reveals three distinct clusters of symptoms, specifically cognitive, emotional, and somatic factors (Potter et al., 2006). This distinction is particularly important as it allows us to investigate whether specific psychiatric groups are more likely to endorse certain symptom clusters, contributing to symptom reporting differences. We undertook a finer analysis of individuals with specific psychiatric diagnoses to assess whether there are significant base rates of PCS present, and whether there are important differences between psychiatric disorders in symptom endorsement. With the above in mind, we set out to more specifically address the following hypotheses:

1. We set out to determine whether non-concussed psychiatric populations report differing levels of PCS symptom endorsement compared to healthy controls. We hypothesize that psychiatric populations will not differ in overall PCS symptom endorsement compared to a mTBI population and a healthy population. That is, that PCS symptoms are not specific to concussion. This is in line with previously highlighted research (Donnell et al., 2012); however, given the noted limitations above, further investigation is warranted.
2. We set out to determine whether individuals with psychiatric diagnoses differentially endorse PCS symptoms.
  We hypothesize that individuals with anxiety disorders and individuals with trauma- and stressor-related disorders will endorse greater somatic symptoms on the RPQ, reflecting the physiological manifestations characteristic of these conditions, as outlined in the literature (Carlehed et al., 2017; Love & Love, 2019; McFarlane et al., 1994; Mohamed et al., 2023; Morrison & Keating, 2001).
  We hypothesize that individuals with somatic symptom and related disorders will report greater emotional and somatic symptoms, given the disorder’s emphasis on worry about illness and associated distress (American Psychiatric Association, 2022; Okur Güney et al., 2019).
  We hypothesize that individuals with complex psychiatric disorders will endorse greater PCS symptoms overall due to the existence of multiple comorbidities and the variety and overlap in disorder symptomatology experienced.

## METHODOLOGY

### Participants

Data for our study was taken from an archival dataset, including a total of 140 participants. Test data was garnered from a private practice in Ontario. Referrals for assessment were varied and include assessment for diagnostic and treatment planning purposes, insurance examinations and medical legal examinations at the request of both defense and plaintiff. Participants underwent a battery of psychological test measures demonstrating internal consistency and reliability, including the *Rivermead Post-Concussion Symptoms Questionnaire,* the *Pain Catastrophizing Scale*, the *Posttraumatic Stress Disorder Checklist for DSM-5*, the *Survey of Pain Attitudes*, the *Depression Anxiety Stress Scale*, and the *Pain Patient Profile*. The diagnostic groups investigated in this study include depressive disorders, anxiety disorders, trauma- and stressor-related disorders, somatic symptom and related disorders, and complex psychiatric disorders, which refer to combinations of three or more psychiatric diagnoses. All diagnoses were made by a registered psychologist (J.F.) holding autonomous practice in the province of Ontario under the College of Psychologists of Ontario. Diagnoses were made based on extensive file review, psychometric testing, clinical interview, and case-conceptualization. Subjects who failed one or more symptom validity measures were not included in our data analyses. Ethics approval was obtained from the University of Toronto Scarborough Campus.

### Demographics

All participants were non-concussed adults between the ages of 18 and 77, referred for independent psychological examination as part of personal injury litigation. The absence of traumatic brain injury was confirmed for all participants by way of ACRM criteria. As shown in Table 1, there were a total of 140 participants with a mean age of 40.6 (*SD* = 14.6), of which 47.1% were male (*n* = 66) and 52.9% were female (*n* = 74). The sample consisted of people born in North America (*n* = 87), the Caribbean and South America (*n* = 17), Asia (*n* = 21), Africa and the Middle East (*n* = 8), and Europe (*n* = 7). All participants indicated that they are comfortable speaking English, and 94 participants selected English as their preferred language. Many participants spoke a second language, including Arabic, Cantonese, Farsi, Filipino, Spanish, and a variety of other languages. Participants with limited English reading and comprehension skills were assisted by a certified court translator. Socioeconomic status was varied and was measured based on a combination of education and employment status.

**Table 1:** Demographic characteristics and Rivermead descriptives by diagnostic group

Diagnostic group	Total sample (*n* = 131)	Trauma- and Stressor-related and Anxiety Disorders (*n* = 51)	Somatic Symptom and Related Disorders (*n* = 18)	Complex Psychiatric Disorders (*n* = 62)
Demographic variables				
Female (%)	52.7	45.0	50.0	59.7
Age (mean [*SD*])	40.6 (14.6)	40.6 (15.1)	44.0 (17.6)	39.7 (13.2)
Education (mean [*SD*])	14.3 (2.7)	14.7 (2.6)	13.2 (2.9)	14.3 (2.8)
RPQ Test Scores (mean [*SD*])				
Total	40.8 (13.7)	32.8 (13.8)	39.3 (11.7)	47.9 (10.1)
Cognitive Cluster	8.3 (3.6)	6.4 (3.3)	8.4 (3.3)	9.8 (2.6)
Emotional Cluster	11.7 (3.9)	9.3 (4.2)	11.9 (3.3)	13.6 (2.5)
Somatic Cluster	19.7 (7.2)	16.2 (7.3)	17.7 (6.8)	23.1 (5.4)

*Note.* Demographic characteristics and mean Rivermead total and symptom cluster scores for the total sample and by diagnostic group.

### Primary Measure of Investigation

Participants completed a variety of questionnaires and tests assessing a combination of psychological and physiological symptoms, many of which are associated with pain. Of interest, the *Rivermead Post-Concussion Symptoms Questionnaire* (RPQ) a self-report measure of PCS, was used to measure the breadth and severity of PCS symptoms. This scale consists of 16 items, evaluating a range of symptoms, including headaches, dizziness, nausea or vomiting, noise sensitivity, sleep disturbances, fatigue or tiring more easily, irritability or easily angered, feeling depressed or tearful, feeling frustrated or impatient, forgetfulness/poor memory, poor concentration, taking longer to think, blurred vision, light sensitivity, double vision, and restlessness (RPQ; King et al., 1995). Symptoms are rated on a scale from 0 to 4, with 0 indicating the symptom was not experienced and 4 indicating it has been a severe problem in the past 24 h, compared to premorbid levels (King et al., 1995). A higher total score on the RPQ is indicative of a greater number of symptoms experienced and greater symptom severity (Potter et al., 2006). This scale was found to have high test–retest reliability in patients with head injuries 7–10 days post-injury (King et al., 1995). Additionally, when tested on patients with head injuries, the RPQ was found to have high interrater reliability for total PCS scores and good reliability for individual PCS items (King et al., 1995). A factor analysis of the RPQ identifies three distinct factors underlying post-concussion symptoms: cognitive, emotional, and somatic factors (Potter et al., 2006), supporting the three-factor structure proposed by Smith-Seemiller et al. (2003), and indicating that these symptom domains can be at least partially differentiated.

## RESULTS

A total of 140 participants were included in the dataset, of which 36.4% (*n* = 51) were classified in the trauma- and anxiety-related disorders group, 13.6% (*n* = 19) in the somatic symptom and related disorders group, 44.3% (*n* = 62) in the complex psychiatric disorders group, and 5.7% (*n* = 8) in the depressive disorders group. Data from the eight participants in the depressive disorders group were excluded due to the small sample size, and one additional participant from the somatic group was excluded due to an incomplete RPQ, resulting in a final analytic sample of 131 participants, of which 52.7% were female. Table 1 presents the demographic characteristics and RPQ scores by diagnostic group.

Tests of normality revealed that the Rivermead total and symptom cluster scores were not normally distributed across diagnostic groups. As a result, nonparametric analyses were conducted to examine between-group differences in RPQ symptom endorsement. A Kruskal-Wallis test revealed a significant effect of diagnostic group on RPQ total (*H*(2) = 33.24, *p* < .001), cognitive cluster (*H*(2) = 31.46, *p* < .001), emotional cluster (*H*(2) = 33.22, *p* < .001), and the somatic cluster (*H*(2) = 27.32, *p* < .001) symptom scores. Post hoc comparisons with Bonferroni adjustment showed that the complex psychiatric disorders group reported significantly greater total, cognitive, emotional, and somatic scores than the trauma/anxiety group (e.g., RPQ total: *p* < .001, *d* = 1.27, OL% = 34.7). The complex group also scored higher than the somatic group on RPQ total (*p* = .032, *d* = .59, OL% = 61.8) and somatic scores (*p* = .009, *d* = .71, OL% = 57.0). Differences between the trauma/anxiety and somatic groups on RPQ total (*p* = .444), cognitive (*p* = .070), emotional (*p* = .089), and somatic (*p* = 1.000) scores, as well as between the somatic and complex groups on cognitive (*p* = .298) and emotional (*p* = .194) scores, were not significant. A summary of the Kruskal-Wallis test results, pairwise comparisons, and corresponding effect sizes and overlap percentages is presented in Table 2.

**Table 2:** Kruskal-Wallis test results for RPQ total and symptom clusters across diagnostic groups

RPQ Test Scores	df	H	*p*	Significant Pairwise Comparisons
Total	2	33.24	< .001	Complex > Trauma/Anxiety (*p* < .001, *d* = 1.27, OL% = 34.7); Complex > Somatic (*p* = .032, *d* = .59, OL% = 61.8)
Cognitive Cluster	2	31.46	< .001	Complex > Trauma/Anxiety (*p* < .001, *d* = 1.25, OL% = 34.7)
Emotional Cluster	2	33.22	< .001	Complex > Trauma/Anxiety (*p* < .001, *d* = 1.06, OL% = 41.1)
Somatic Cluster	2	27.32	< .001	Complex > Trauma/Anxiety (*p* < .001, *d* = 1.07, OL% = 41.1); Complex > Somatic (*p* = .009, *d* = 0.71, OL% = 57.0)

*Note.* Kruskal–Wallis test results and Bonferroni-adjusted pairwise comparisons for RPQ total and symptom cluster scores across diagnostic groups. OL% = overlap percentage.

We additionally compared RPQ total scores from the current psychiatric sample to reference values drawn from existing literature, including a multicultural healthy sample (Zakzanis & Yeung, 2011) and a TBI sample (Zeldovich et al., 2022). As shown in Table 3, all psychiatric diagnostic groups, as well as the entire psychiatric sample, reported markedly higher RPQ total scores relative to both the healthy and TBI samples.

**Table 3:** Mean RPQ total scores compared to a healthy control sample and a TBI sample

Group	*n*	Mean RPQ Total (*SD*)
Trauma- and Anxiety Disorders	51	32.75 (13.81)
Somatic Symptom and Related Disorders	18	39.33 (11.72)
Complex Psychiatric Disorders	62	47.85 (10.09)
Entire Psychiatric Sample	131	40.80 (13.74)
Healthy Controls (Zakzanis & Yeung, 2011)	151	12.74 (10.00)
mTBI Sample (Zeldovich et al., 2022)	223	15.10 (12.70)

*Note.* Mean RPQ total scores across diagnostic groups compared to healthy controls and a TBI sample.

## DISCUSSION

The present study is the first to demonstrate that non-concussed psychiatric populations endorse significant levels of PCS-like symptoms, with particularly elevated endorsement among individuals with multiple comorbidities. The mean RPQ total score across the entire psychiatric sample was 40.8, with the trauma- and anxiety-related disorders group endorsing the lowest overall symptom burden and complex psychiatric disorder group endorsing the highest. These results support the hypothesis that PCS-like symptoms are not specific to concussion and are also endorsed among psychiatric populations, congruent with prior research showing high base rates of such symptoms in healthy groups (Wang et al., 2006; Zakzanis & Yeung, 2011). Notably, symptom endorsement was particularly elevated among those with multiple comorbidities, highlighting the influence of psychiatric factors on concussion symptom burden.

In terms of our second hypothesis, Kruskal–Wallis analyses revealed that symptom cluster endorsement varied significantly across diagnostic groups, though not always in the ways predicted. Contrary to our hypotheses, individuals with trauma- and anxiety-related disorders did not endorse greater somatic symptoms, and those with somatic symptom and related disorders did not report elevated emotional or somatic symptoms relative to the other diagnostic groups. Rather, it was the complex psychiatric disorders group that consistently reported the highest symptom burden across RPQ domains, supporting the hypothesis that multiple comorbidities are associated with greater overall symptom endorsement. Overlap percentages (OL%) ranged from the mid-30s to low-60s, illustrating varying degrees of separation between diagnostic groups. OL% reflects the degree of overlap in score distributions between groups, making it an important indicator for interpreting the practical significance of test differences (see Zakzanis, 2001).

Taken together, our results illustrate patterns of PCS-like symptom endorsement across psychiatric populations. While hypotheses regarding the trauma/anxiety and somatic symptom groups were not supported, the elevated symptom endorsement observed in the complex psychiatric disorders group emphasizes the role of psychiatric comorbidity in concussion symptom burden. These results reinforce that PCS-like symptoms are not unique to mTBI and highlight concerns about diagnostic specificity, as psychiatric symptoms can closely resemble those attributed to concussion. Such overlap in symptoms increases the risk of misattribution, where common psychiatric manifestations (e.g., anxiety, fatigue, and concentration difficulties) may be mistakenly linked to neurological injury. This aligns with the broader literature on expectation as etiology and nocebo effects, which emphasizes that symptom interpretation is often shaped by cognitive and emotional factors rather than injury-specific pathology (McCrea, 2007b; Mittenberg et al., 1992; Polich et al., 2019).

Comparisons to external samples further highlight the magnitude of PCS-like symptom endorsement in psychiatric groups. When comparing the mean RPQ total scores from our psychiatric sample to previously published data, all psychiatric groups demonstrated substantially higher scores than both the multicultural healthy control group (Zakzanis & Yeung, 2011) and the TBI sample (Zeldovich et al., 2022). The complex psychiatric disorder group reported the greatest symptom burden, with mean total scores more than double those of both the healthy and TBI samples. These findings demonstrate that PCS-like symptoms are not only present across psychiatric populations but, strikingly, are endorsed at higher levels than by individuals with documented neurological trauma, where one would expect the highest scores.

This raises a critical question: why would psychiatric groups, particularly those with multiple comorbidities, endorse more concussion-like symptoms than individuals with a history of neurological trauma? One explanation lies in the nonspecific nature of PCS symptomatology. Complaints such as fatigue, poor concentration, and headaches, are not specific to brain injury and are core symptoms of many psychiatric disorders. A second explanation is the role of anxiety, which was a common underlying theme across the diagnostic groups examined. Physiological hyperarousal associated with anxiety can produce somatic and cognitive symptoms that resemble concussion-like symptoms, and when combined with attentional bias towards bodily sensations, may increase the likelihood that ordinary fluctuations in health are experienced and reported as symptoms. In individuals with complex psychiatric disorders, these processes may interact in additive ways, creating a cumulative symptom burden that exceeds what is observed in neurologically injured groups.

These proposed mechanistic processes are consistent with integrative models of medically unexplained symptoms, which suggest that somatic complaints emerge from the interaction between bottom-up bodily sensations and top-down expectations (Sauer et al., 2023). Somatosensory amplification and cognitive biases may further reinforce this process, resulting in symptom reports that appear neurologically driven but in fact reflect psychiatric processes (Sauer et al., 2023). This interpretation aligns with evidence that expectation, misattribution, and nocebo effects can sustain or exacerbate symptoms even in the absence of brain injury (e.g., Mittenberg et al., 1992). Taken together, the present findings suggest that psychiatric symptoms, particularly in the presence of multiple comorbidities, may play a greater role in PCS-like symptom endorsement than neurological trauma itself. In this sense, the irony reflected in the title becomes clear: while concussion-like symptoms may resemble those seen after mTBI, their presence does not necessarily indicate neurological injury. In other words, not every four-legged animal is a dog, and not every presentation of PCS-like symptoms can be attributed to mTBI.

Several limitations should be considered when interpreting these findings. The archival nature of the dataset restricted control over diagnostic distribution, leading to uneven group sizes and a small sample for the somatic symptom disorders group, which may have limited the ability to detect more subtle effects. In addition, comparisons to external healthy and TBI samples relied on previously published data, which may differ in sampling methods or assessment conditions, limiting direct comparability. These limitations highlight the need for future research to examine PCS-like symptoms in larger and diagnostically balanced samples. Additionally, studies that incorporate neurobiological and psychophysiological measures would also help to test proposed mechanisms such as hyperarousal and attentional bias, providing a more direct link between psychiatric burden and concussion-like symptomatology.

## Notes

Author Note: A portion of these analyses were presented as a poster at the Canadian Psychological Association’s 2025 National Convention, St. John’s, Newfoundland, Canada. The authors declare no conflicts of interest.
